# Choroidal metastasis as the first manifestation of renal pelvis carcinoma: A case report

**DOI:** 10.1097/MD.0000000000042268

**Published:** 2025-04-25

**Authors:** Songtian Che, Ranwei Li, Xu Wang, Guiyun Wang, Jinfeng Cao

**Affiliations:** a Department of Ophthalmology, The Second Hospital of Jilin University, Changchun, China; b Department of Urology, The Second Hospital of Jilin University, Changchun, China.

**Keywords:** case report, choroidal metastasis, renal pelvis carcinoma, retinal detachment

## Abstract

**Rationale:**

Choroidal metastasis is an uncommon event, especially as the first sign of renal pelvis carcinoma (RPC), a rare subtype of upper tract urothelial carcinoma (UTUC). While the majority of choroidal metastases originate from primary cancers like breast or lung, those arising from urologic cancers are extremely rare. This article describes a case of RPC where the first clinical sign was a choroidal mass.

**Patient concerns:**

A 51-year-old female presented with a 2-week history of decreased vision in her left eye (OS). She reported no prior history of malignancy or significant family history of cancer. Examination revealed retinal detachment and a reddish-white, dome-shaped choroidal lesion. Multimodal imaging, including indocyanine green angiography, Doppler ultrasound, optical coherence tomography confirmed the metastatic nature of the mass.

**Diagnoses:**

Magnetic resonance imaging, computed tomography and whole-body bone scintigraphy with 99mTc-MDP detected multiple metastases to the brain, lungs, and bone, with primary RPC in the left renal pelvis. These findings led to the diagnosis of metastatic RPC.

**Interventions:**

Given the advanced disease stage and poor prognosis, the patient declined invasive treatments such as biopsy or systemic chemotherapy.

**Outcomes:**

Two weeks after diagnosis, the patient succumbed to rapid disease progression.

**Lessons:**

This is a unique case of RPC presenting with tetra-organ metastases involving the lung, bone, brain, and choroid. It underscores the need for a comprehensive systemic evaluation in patients with unexplained choroidal masses, as these may be indicative of an underlying, often asymptomatic, systemic malignancy. Further therapeutic studies are essential to explore effective management strategies and improve outcomes for similar patients with RPC.

## 1. Introduction

Choroidal metastasis, though relatively rare, is a significant ocular manifestation of systemic malignancies caused by hematogenous spread. It is mostly associated with primary cancers of the breast and lung; however, metastases from other origins, including urologic malignancies, have been documented, albeit rarely.^[1]^ Renal cell carcinoma (RCC) is the most common urologic cancer to metastasize to the choroid, while metastasis of renal pelvis carcinoma (RPC) to the eye is extremely rare, with only 2 cases reported in the literature.^[2,3]^ Once choroidal metastasis is diagnosed, it usually indicates widespread metastases and carries a poor prognosis. Here, we present the case of a female patient with unilateral retinal detachment and choroidal metastasis as the initial manifestation of previously undiagnosed RPC, accompanied by metastases to the brain, bone, and lung.

## 2. Case presentation

**Figure 1.**
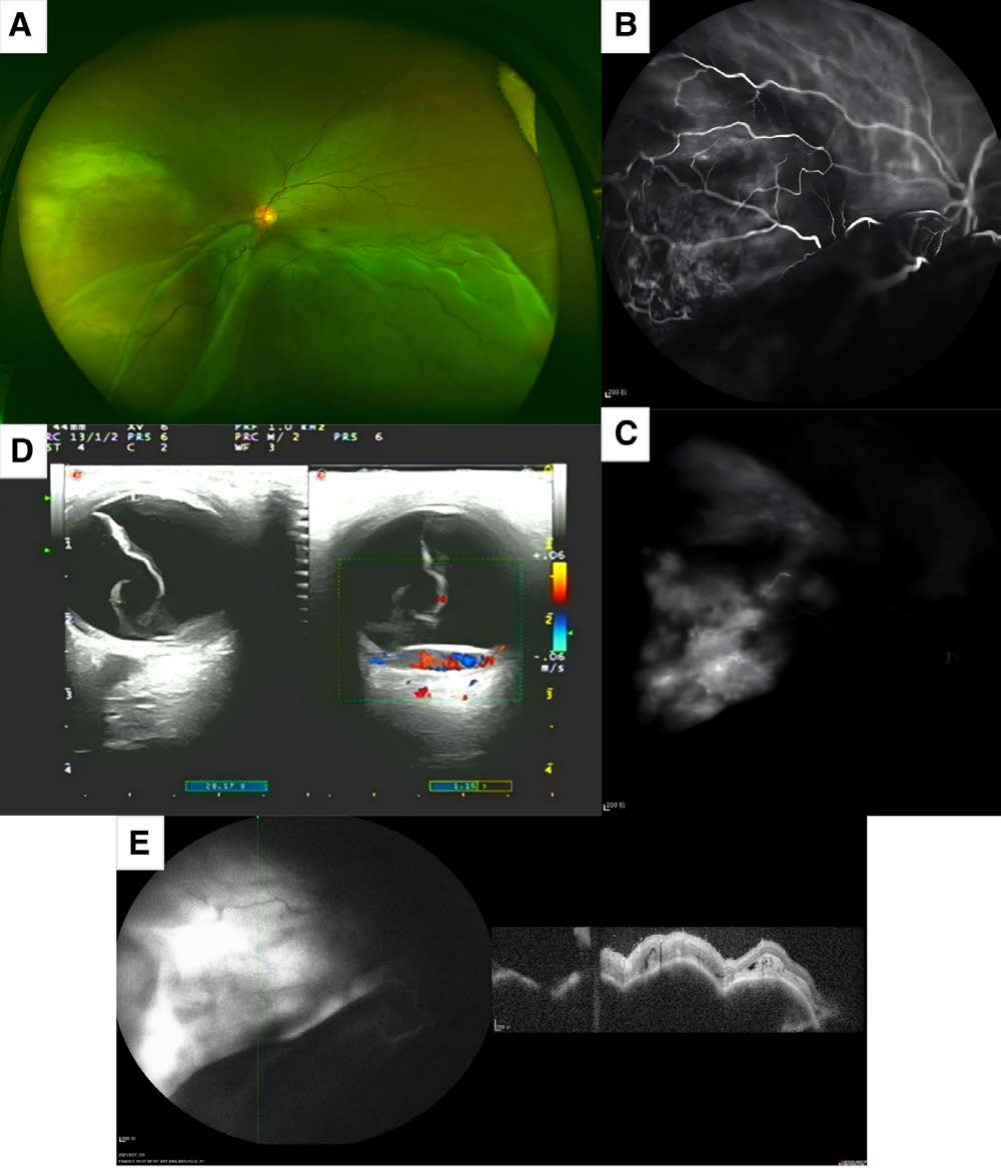
Fundoscopy, ICGA, Doppler ultrasound and OCT examinations of the patient at presentation. Ultra-wide-field fundus photography showed inferior retinal elevation and a dome-shaped, reddish-white choroidal mass nasal to the optic disc (A). ICGA showed early-stage mottled hyper- and hypofluorescence in the nasal region (B), with late-stage diffuse hyperfluorescence interspersed with areas of hypofluorescence, accompanied by leakage (C). Doppler ultrasound identified a nasal choroidal mass with a basal diameter of 14.0 mm and a thickness of 0.42 mm, exhibiting abundant blood flow signals (D). OCT revealed choroidal elevation, significant compression of the choriocapillaris, and disruption of the photoreceptor layer (E). ICGA = indocyanine green angiography, OCT = optical coherence tomography.

A 51-year-old female presented with a 2-week history of decreased vision in her left eye (OS) in August 2021. She had no significant medical or family history of cancer. The best corrected vision acuity was 20/20 in the right eye and hand motion at 20 cm in the OS. Fundus examination of OS revealed inferior retinal detachment without a break, along with an amelanotic, reddish-white, dome-shaped choroidal lesion nasal to the optic disc (Fig. 1A). Indocyanine green angiography showed diffuse hyper- and hypofluorescence at early stage (Fig. 1B) and late stage (Fig. 1C). Doppler ultrasound detected a nasal choroidal mass measuring 14.0 mm in basal diameter and 0.42 mm in thickness, with abundant blood flow signals (Fig. 1D). Optical coherence tomography indicated choroidal elevation, extreme compression of the choriocapillaris, and disruption of the photoreceptor layer (Fig. 1E).

**Figure 2.**
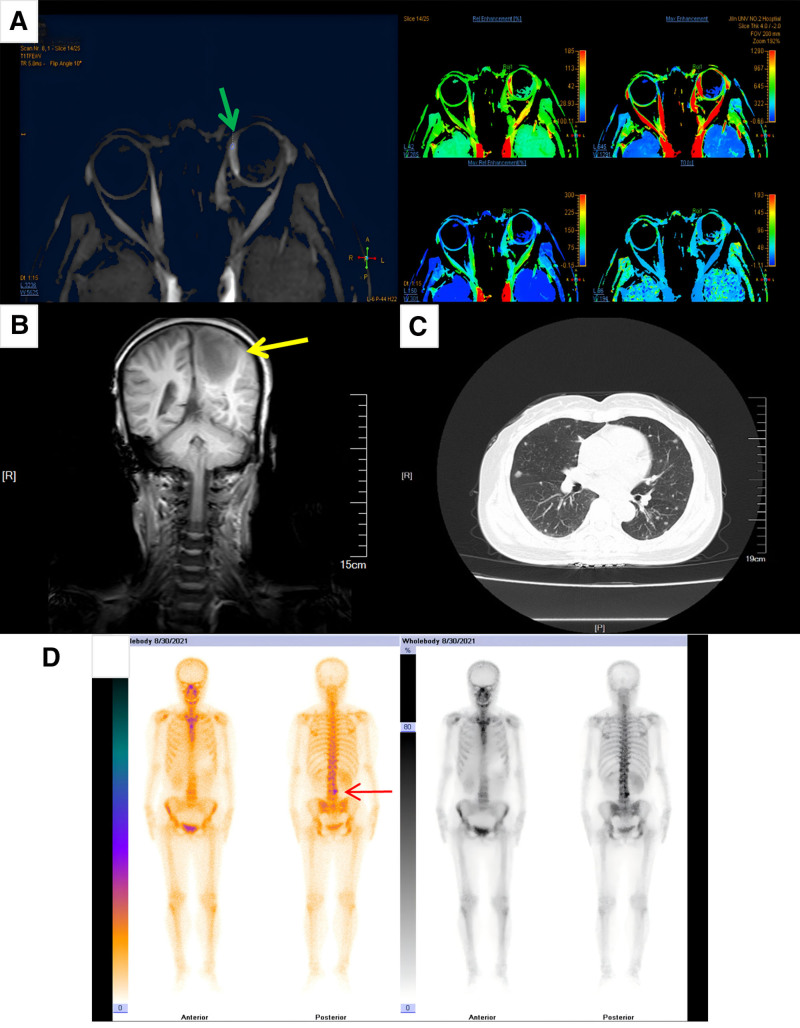
Multiple examinations showed malignancies in the orbit, brain, lung and bone. MRI presented a nasal choroidal tumor (A, green arrow) and an intracranial mass in the left parietal lobe (B, yellow arrow). Chest CT demonstrated multiple pulmonary nodules (C). Whole-body bone scintigraphy with 99mTc-MDP showed increased radiotracer uptake in the L4 vertebra (D, red arrow). CT = computed tomography, MRI = magnetic resonance imaging.

**Figure 3.**
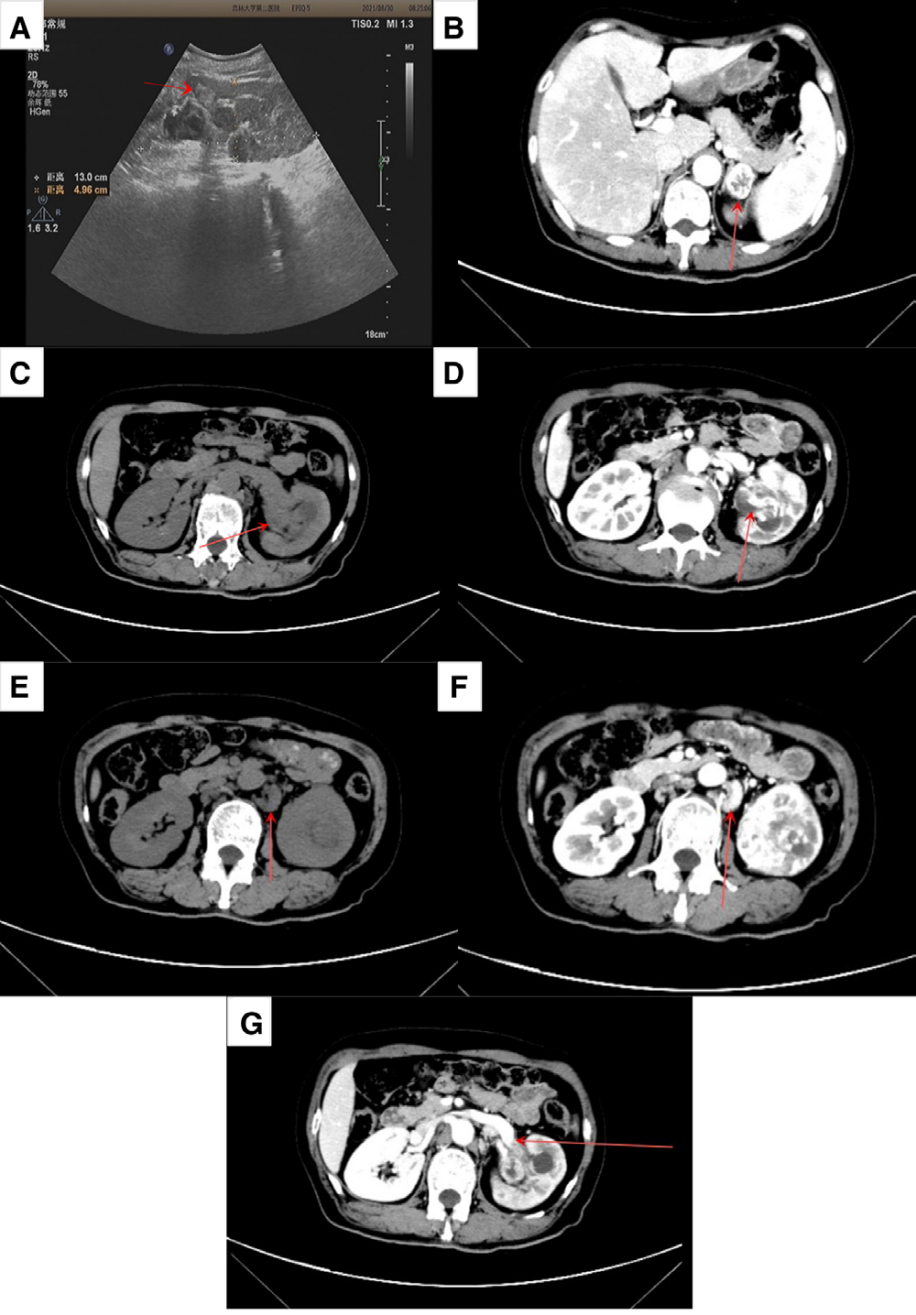
Renal pelvis carcinoma identified by ultrasound and CT scan. Ultrasound revealed the neoplasm in the left renal pelvis (A). Contrast-enhanced abdominal CT showed left adrenal involvement in the arterial phase (B), a tumor in the left renal pelvis in both the non-contrast phase (C) and arterial phase (D), and adjacent lymph node involvement in the non-contrast phase (E) and arterial phase (F). Additionally, a tumor thrombus was identified in the renal vein during the venous phase (G). The tumor is indicated by the red arrow. CT = computed tomography.

Further investigations included magnetic resonance imaging, which showed tumors in the nasal choroid (Fig. 2A) and left parietal lobe (Fig. 2B), and chest computed tomography (CT), which demonstrated multiple lung nodules (Fig. 2C). Increased radiotracer uptake is observed in the L4 vertebra using whole-body bone scintigraphy with 99mTc-MDP (Fig. 2D). Ultrasound detected the neoplasm in the left renal pelvis (Fig. 3A), and contrast-enhanced abdominal CT confirmed a tumor in the left renal pelvis, along with adjacent lymph node involvement, adrenal gland involvement, and tumor thrombus in the renal vein (Fig. 3B–F). Blood tests showed normal liver and kidney function, with mild anemia (hemoglobin 100 g/L), and urinalysis showed 3-point 4 red blood cells per high power field.

Based on the imaging findings and clinical presentation, a diagnosis of choroidal, brain, lung, and bone metastases originating from RPC was made. The patient hesitated to proceed with a biopsy or treatment and died 2 weeks after the diagnosis of choroidal metastasis due to rapid disease progression.

## 3. Discussion

The choroid, a highly vascularized layer of the eye, is a rare but significant site of systemic metastasis. Most choroidal metastases originate from breast or lung cancers, which tend to spread hematogenously.^[4]^ Metastasis from urologic cancers, particularly RPC, is extremely rare. RPC is a subtype of upper tract urothelial carcinoma (UTUC) and accounts for only 5% to 10% of urothelial cancers, with bladder cancer making up the majority.^[5]^ UTUCs, including RPC, typically present with advanced disease due to vague and nonspecific symptoms such as hematuria, flank pain, or urinary obstruction.^[6]^ Imaging techniques like CT urography play a crucial role in diagnosing and staging these tumors, with radical nephroureterectomy being the standard treatment. However, prognosis is generally poor compared to bladder cancer due to more advanced presentation at diagnosis.^[7]^

This case is unique in its initial presentation of RPC through ocular symptoms, specifically retinal detachment and a choroidal mass, without other systemic indications. Unlike RCC, which often metastasizes via the bloodstream, UTUCs generally spread through the lymphatic system, which may explain the rarity of ocular metastasis from RPC.^[8]^ The precise mechanism behind hematogenous spread to the choroid in this case remains unclear. The rapid progression and widespread metastases to the brain, bone, and lung are consistent with the poor prognosis associated with late-stage UTUCs.

A Surveillance, Epidemiology, and End Results-based study shows that the primary metastatic sites of RCC are the lungs, bones, liver, and distant lymph nodes, with brain metastasis being relatively rare, accounting for only 2.8% of cases among 424 metastatic RCC patients. Additionally, the tetra-organ metastatic pattern was observed in only 2.6% of patient.^[9]^ To the best of our knowledge, our case is the first reported case of RPC presenting with tetra-organ metastases involving the lung, bone, brain, and choroid.

The limitation of this case report is the absence of histopathological confirmation, indicating our diagnosis remains presumptive rather than definitive. Although imaging modalities (CT, MRI and ultrasound) strongly support RPC and metastases, the patient’s deteriorating condition and her refusal of invasive procedures rendered biopsy or surgical confirmation unfeasible. One may question the accuracy of the diagnostic imaging modalities, however, it is important to note that CT imaging has demonstrated a high accuracy rate (97%) in diagnosing UTUC compared with histopathology findings, especially in tumors of the ureter and renal pelvis, as shown in a study of 275 patients.^[10]^

In conclusion, this rare case of choroidal metastasis as the initial presentation of RPC highlights the diagnostic complexity of metastatic disease with unusual symptoms. It underscores the importance of comprehensive ocular and systemic evaluations in patients with unexplained retinal detachment or choroidal mass.

## Acknowledgments

We thank Dr Huiying Wu for interpreting the results of ultrasound.

## Author contributions

**Conceptualization:** Songtian Che, Guiyun Wang, Jinfeng Cao.

**Data curation:** Jinfeng Cao.

**Formal analysis:** Ranwei Li.

**Investigation:** Songtian Che, Xu Wang.

**Resources:** Guiyun Wang.

**Writing – original draft:** Jinfeng Cao.
